# Construction of a home-based social support service content framework for Chinese patients with chronic heart failure based on Maslow's hierarchy of needs

**DOI:** 10.3389/fmed.2026.1953127

**Published:** 2026-09-21

**Authors:** Haoxin Tian, Haotian Zhang, Yuntao Yu, Enyuan Yan, Jie Chen, Tiantian Liu, Shaoye Gong, Yanyan Fan

**Affiliations:** School of Nursing, Shandong Medical and Pharmaceutical University, Shandong, China

**Keywords:** China, chronic heart failure, delphi method, home-based social support, social support services

## Abstract

**Objective:**

This study aimed to construct a home-based social support service content framework for Chinese patients with chronic heart failure (CHF), so as to provide a theoretical and content foundation for addressing their home-based social support needs.

**Methods:**

Guided by Maslow's hierarchy of needs, a home-based social support service content framework for Chinese CHF patients was developed through a combination of literature review, semi-structured interviews, and the Delphi expert consultation method. The weights of the identified indicators were subsequently determined using the Analytic Hierarchy Process (AHP).

**Results:**

A total of 7 primary-level, 17 secondary-level, and 50 tertiary-level indicators were initially derived from the literature review and semi-structured interviews. In the two Delphi rounds, the effective response rates were both 100%, with expert suggestion rates of 53.4% and 20.0%, and authority coefficients of 0.872 and 0.881, respectively. Kendall's W values for the two rounds were 0.306 and 0.310 (both *P* < 0.001). Following two rounds of expert consultation, the final framework comprised 7 primary-level, 18 secondary-level, and 58 tertiary-level indicators.

**Conclusions:**

The developed home-based social support service content framework for Chinese CHF patients demonstrates satisfactory scientific rigor and reliability, and may serve as a useful reference for healthcare providers in addressing the home-based social support needs of this population.

## 1 Introduction

Chronic heart failure (CHF) represents the severe and terminal stage of various cardiovascular diseases, characterized by high recurrence rates and substantial disease burden (1). Globally, over 64 million individuals are affected by CHF, posing significant strain on healthcare systems worldwide (2). In China, approximately 14.3 million patients live with CHF, generating considerable demand for medical services (3, 4).

Healthy aging extends beyond disease-free survival to encompass the comprehensive maintenance of functional capacity, emotional well-being, social participation, and overall quality of life (5). For older adults with chronic conditions, particularly those with CHF, these dimensions are inherently interconnected and mutually influential. Social networks, physical activity and social engagement, and activities of daily living have been identified as modifiable facilitating factors that promote proactive healthy aging (6). Consequently, needs assessment in CHF patients should not be confined to clinical parameters but must also incorporate psychosocial and lifestyle-related dimensions that affect long-term quality of life.

Research indicates that social support services play a critical role in enhancing self-management capacity and improving quality of life among CHF patients (7, 8). However, current satisfaction rates with home-based social support services for CHF patients range from 40.0% to 60.0%, with service gaps exceeding 50.0% (9). Notably, unmet needs are particularly pronounced in psychological and emotional support, social belonging, and self-actualization (10). Furthermore, information asymmetry and inaccessibility of service resources are major contributors to low utilization rates and inefficient resource allocation within existing support systems (11).

Maslow's hierarchy of needs is a classic theoretical model encompassing multiple levels of human needs, including physiological, safety, love and belonging, esteem, cognitive, aesthetic, and self-actualization, thereby providing a relatively comprehensive coverage of fundamental individual needs (12). This theory has been applied to social support needs assessment and service design among populations such as lung cancer survivors, persons with disabilities, and hemodialysis patients, demonstrating sound theoretical fit and practical guiding value (13–15). Alternative models, such as Orem's self-care theory, emphasize patients' self-care capacity, while the Chronic Care Model focuses on health system reorganization, each valuable in its own right yet relatively narrow in scope (16, 17). We selected Maslow's framework primarily because its multi-tiered structure directly corresponds to the need dimensions addressed in this study. More importantly, it exhibits intrinsic methodological compatibility with the Analytic Hierarchy Process (AHP). As a multi-criteria decision-making method, AHP allows for weight assignment to elements within a hierarchical structure, thereby complementing Maslow's tiered need architecture. In the context of chronic illness, physiological, psychological, cognitive, and social needs often coexist rather than emerge in a strict sequential order. However, unlike a rigid stage-wise interpretation of Maslow's theory, the AHP approach adopted in this study does not presuppose a linear progressive sequence of needs; rather, it permits simultaneous weighting and comprehensive evaluation of multiple coexisting needs. This methodological choice enables us to retain the theoretical structure while more faithfully approximating the genuine complexity of clinical practice.

Existing studies on social support needs among patients with CHF still have room for improvement regarding both the systematicity of theoretical frameworks and the comprehensiveness of need dimensions, and relevant research targeting the Chinese patient population remains relatively limited. To address these gaps, this study, grounded in Maslow's hierarchy of needs, constructs a home-based social support service content framework for Chinese CHF patients. The framework integrates both patient and family caregiver perspectives into a unified needs system, incorporates clinical indicators and psychosocial needs into a coherent hierarchical structure for joint analysis, and employs the AHP to empirically assign weights to each need dimension, thereby providing multidisciplinary care teams with actionable prioritization criteria. This study aims to advance the conceptual understanding of comprehensive needs assessment in CHF at the theoretical level, while offering practical references for the design of precision-targeted home-based social support services, and contributing insights to improve the precision and accessibility of such service provision globally.

## 2 Methods

### 2.1 Establishment of the research group

The research group comprised seven members: one cardiovascular clinical specialist, three cardiovascular nursing specialists, one chronic disease management expert, and two registered nurses. The group's primary responsibilities included preliminary formulation of relevant indicators, design and administration of expert consultation questionnaires, and statistical analysis and discussion of consultation feedback.

### 2.2 Theoretical framework

Maslow's hierarchy of needs, proposed by American psychologist Abraham Harold Maslow from the perspective of human motivation, encompasses seven tiers (15). The operational definitions of the specific concepts adopted in this study are presented in Table 1.

**Table 1:** Operational definitions of maslow's hierarchy of needs dimensions.

Need Level	Conceptual Definition in This Study
A Physiological needs	Support to address patients' requirements for daily care, skilled nursing, nutritional adequacy, and individualized exercise and rehabilitation programs.
B Safety needs	Support to ensure home environmental safety, continuity of healthcare delivery, and financial protection through medical insurance coverage.
C Love and belonging needs	Support to sustain patients' sense of belonging and relational connectedness through family caregiving dynamics, emotional bonding, and social engagement.
D Esteem needs	Support to affirm patients' status as autonomous and valued individuals, with emphasis on protecting their privacy, decision-making autonomy, and self-esteem.
E Cognitive needs	Support to facilitate patients' comprehension of disease-related information, development of self-management competencies, and capacity to obtain and utilize health-related resources.
F Aesthetic needs	Support to accommodate patients' physical and psychological preferences for personal presentation, comfortable clothing, and a soothing and dignified care environment.
G Self-actualization needs	Support to assist patients in redefining personal meaning and fulfilling their potential for value realization despite the constraints of chronic illness.

### 2.3 Comprehensive literature review

#### 2.3.1 Literature search strategy

A systematic literature search was conducted across multiple Chinese and international databases, including PubMed, Web of Science, Cochrane, EMBASE, CINAHL, China National Knowledge Infrastructure (CNKI), WanFang Data, Weipu Data (VIP), and the China Biology Medicine Database (CBM). Search terms comprised home care, home rehabilitation, home management, chronic heart failure, heart failure patients, social support services, social support contents, emotional support, informational support, and instrumental support. A combination of MeSH terms and free-text words was employed, with the search timeframe spanning from database inception to September 2025. The detailed search strategy is presented in Supplementary Table S1.

The inclusion criteria were established following the PCC framework (Participants, Concept, Context): a) Participants (P): community-dwelling patients aged ≥18 years with a confirmed diagnosis of CHF; b) Concept (C): home-based support service contents with supportive attributes, provided by formal or informal caregivers, that address the needs of CHF patients, encompassing emotional support, informational support, instrumental support, and social participation-related support; c) Context (C): home, community, and primary healthcare settings. The exclusion criteria were: a) document types restricted to conference papers, notices and announcements, newspapers, and short reports; b) articles with incomplete content or unavailable full text; c) duplicate publications; d) articles published in languages other than Chinese or English.

#### 2.3.2 Data extraction and synthesis

The quality of included studies was assessed using the QQM Checklist (18), a tool applicable to qualitative, quantitative, and mixed-methods research. All studies were evaluated against eight generic criteria: Setting, Recruitment, Administration, Consent, Ethics, Sample, Achieved Sample, and Sample Size. For qualitative studies, six additional indicators were applied: Instrument content, Instrument content rationale, Instrument example, Analysis method, Analysis example, and Reflexivity. For quantitative cross-sectional designs, four supplementary items, MissingD, MissingS, Valid, and Descriptives, were assessed, with longitudinal or repeated-measures designs further requiring Attrition and RepeatR. Mixed-methods studies were evaluated using a combination of the above criteria. Each indicator was scored as follows: No = 1 point, Partially = 2 points, Yes = 3 points. Studies scoring ≥80% of the total possible score were classified as high quality, 51%–79% as moderate quality, and <50% as low quality.

Two researchers independently read the full texts of the included studies and extracted explicitly mentioned home-based social support service contents based on the PCC framework. Extracted information included authors, year, country, study type, and specific support service items. Following extraction, the two researchers cross-checked their results; in cases of disagreement, a third researcher was consulted to reach consensus through discussion. All extracted support service items were then consolidated by category and deduplicated conceptually, resulting in a preliminary literature-based indicator framework for home-based social support services.

### 2.4 Semi-Structured interviews

#### 2.4.1 Interview procedure

Purposive sampling was employed to select patients with chronic heart failure and their primary family caregivers from a tertiary hospital in Shandong Province and its affiliated community health service centers for semi-structured interviews. The inclusion criteria for patients were: a) meeting the diagnostic criteria for chronic heart failure (19); b) aged ≥18 years; c) NYHA functional class I–IV with non-initial diagnosis; d) having normal verbal expression and comprehension abilities, and providing voluntary informed consent to participate. The exclusion criteria for patients were: a) disease duration <3 months; b) coexistence of other severe conditions, such as severe hepatic or renal failure.

The inclusion criteria for caregivers were: a) aged ≥18 years; b) assuming primary daily care responsibilities for the patient for ≥3 months; c) having normal communication abilities and providing voluntary informed consent to participate. The exclusion criteria for caregivers were: a) paid professional caregivers; b) anticipated termination of caregiving role or loss to follow-up within the next 6 months; c) concurrent participation in other interventional studies.

The semi-structured interviews were conducted in accordance with the COREQ guidelines (20). Interviews were carried out by two registered nurses from the research team, both holding master's degrees and having undergone specialized training prior to the interviews to ensure interview quality and data collection integrity. The interviewers familiarized themselves with the interview guide and participant characteristics before conducting pilot interviews to refine the guide. The final interview guide is presented in Supplementary Table S2. After obtaining signed informed consent from participants, the interviewers conducted semi-structured interviews in hospital teaching rooms or community health facilities. Interviews were terminated when no new information emerged, indicating data saturation. Each interview lasted approximately 30 min and was recorded with both written notes and audio recording. Based on the collated interview data, the interviewing process was concluded once no new themes were identified. No third-party individuals were present during the interviews. Finally, content analysis was employed to analyze the interview data and distill thematic findings.

#### 2.4.2 Data analysis

The research team maintained continuous reflective records throughout the entire study process. Two interviewers completed interview memos within 24 h following each interview, documenting their own feelings, presuppositions, and potential biases. These memos were reviewed jointly with senior researchers during weekly team meetings to identify and bracket the potential influence of researchers' preconceptions on data collection and interpretation. This reflexive process was sustained throughout all stages of coding and thematic development.

All interview audio recordings were transcribed verbatim within 24 h of completion and returned to participants for confirmation. Following participant verification, two researchers independently coded the interview data using content analysis. Specifically, the two researchers read the transcripts line by line and flagged all statements related to social support needs or service contents. Initial coding was then performed on the flagged content, assigning identical codes to expressions with similar meanings. After coding completion, the codes were categorized and synthesized, and themes were distilled, with sub-themes further refined under each dimension to generate preliminary indicator items for the interview-based dimension. Any discrepancies in coding were resolved through discussion until consensus was reached, or by adjudication from a third researcher.

### 2.5 Indicator item integration and preliminary statistics

In this study, data obtained from the literature review and semi-structured interviews were collated and subjected to preliminary statistical treatment to generate an initial indicator pool. Indicator items extracted from the literature review and thematic items distilled from the semi-structured interviews were compared, merged, and deduplicated to form the “Initial Indicator Pool of Home-Based Social Support Services for Patients with Chronic Heart Failure”. Frequency statistics were employed to count the number of mentions for each coding node in the interview data, and items mentioned at least twice were retained; items appearing in only one included article and lacking empirical support were flagged and provisionally excluded (21). Finally, following the preliminary logic of the Content Validity Index (CVI), five members of the research group internally rated each item in the initial indicator pool for relevance (1 = not relevant, 4 = highly relevant) and importance (1 = not important, 4 = very important). The mean ± standard deviation and the item-level content validity index (I-CVI) were calculated for each item. Items with an I-CVI ≥ 0.78 and a mean importance score ≥3.5 were retained to form the first-round draft of the expert consultation questionnaire.

### 2.6 Delphi expert consultation

#### 2.6.1 Selection of consultation experts

Purposive sampling was employed to select experts for this study. Initially, the research team identified potential experts within tertiary hospitals, schools of public health at universities, and community health management institutions in Shandong Province based on disciplinary categories, and formal invitations were extended through the human resources or research administration departments of these institutions. This was supplemented by a literature-based retrieval strategy-specifically, by searching for academic publications in the fields of chronic heart failure care and chronic disease management, the team additionally identified eligible scholar-type experts, to whom invitations were sent via publicly available institutional contact information. The inclusion criteria for experts were: a) at least ten years of work experience in chronic heart failure treatment, nursing, or management; b) a bachelor's degree or above; c) holding the academic rank of associate professor or an equivalent or higher professional title; d) voluntary participation in this study. The exclusion criteria were: a) having been away from frontline practice in clinical care, nursing, chronic disease management, or social work for more than two years, and no longer directly engaged in relevant activities; b) inability to complete the two rounds of expert consultation as scheduled due to personal health reasons, work schedule conflicts, or time constraints.

#### 2.6.2 Development of the expert consultation questionnaire

The questionnaire comprised four sections: a) an introduction outlining the study theme, objectives, significance, and instructions for completion; b) a demographic information form for experts, covering name, age, gender, educational background, professional title, position, years of working experience, specialty area, and contact information; c) the main body of the questionnaire, consisting of the indicator pool consultation form for the home-based social support service content framework for patients with chronic heart failure. Experts rated the importance of each indicator at all levels using a 5-point Likert scale, ranging from “very unimportant” to “very important”, with corresponding scores of 1–5. Experts assigned importance scores to each level of indicators and provided comments on suggested modifications, deletions, or additions; and d) experts' familiarity with the research content and their judgment basis.

#### 2.6.3 Implementation of expert consultation

A total of 15 experts were ultimately enrolled, representing six disciplinary fields: clinical medicine, nursing, public health, health administration, chronic disease management, and social work (22). All experts were based in China. The research team conducted two rounds of Delphi expert consultation from December 2025 to January 2026. In the first week, questionnaires were distributed to experts via email, with a request for responses within one week. After the first-round questionnaires were collected, the research team discussed the expert opinions and statistical results within two weeks, revising the indicators strictly according to the inclusion criteria, coefficient of variation <0.25 and mean importance score >3.5, and incorporating expert comments to develop the second-round consultation questionnaire. Following distribution of the second-round questionnaire, experts were again asked to respond within one week. After the questionnaires were collected, the remaining time was devoted to statistical analysis and synthesis of expert opinions, culminating in the final version of the indicator framework for home-based social support services for Chinese patients with chronic heart failure.

#### 2.6.4 Data collation and statistical analysis

After the consultation questionnaires were collected, two researchers independently entered and verified the data using Excel 2019, followed by statistical analysis using SPSS 29.0. Expert positivity was calculated as the questionnaire response rate using the formula: Expert positivity coefficient (%) = (number of valid questionnaires returned in each round/total number of questionnaires distributed in that round) × 100%. A response rate ≥70% indicated a high level of expert engagement. Expert authority was evaluated using the expert authority coefficient (Cr), which is the mean of the expert familiarity score (Cs) and the expert judgment basis score (Ca). Both Cs and Ca were self-rated by experts on a scale of 0–1. A Cr ≥ 0.7 indicated greater reliability of the consultation results.

The degree of consensus among expert opinions was measured using the coefficient of variation (CV) and Kendall's coefficient of concordance (Kendall's W). A CV < 0.25 indicated that expert opinions tended to be consistent. Kendall's W ranges from 0 to 1; the closer the value is to 1, the higher the degree of consensus among experts, with *P* < 0.001 indicating reliable results (23). Yaahp 12.4 software was used to construct the hierarchical structure model, and the Saaty scale was employed to build the judgment matrix, from which the weight and composite weight of each indicator were calculated, yielding the consistency ratio (CR) (24).

### 2.7 Statistical analysis

Data were entered using Excel 2019 and analyzed using SPSS 29.0. Categorical variables were presented as frequencies (*n*) and percentages (%), with between-group comparisons performed using the chi-square test. Continuous variables following a normal distribution were expressed as mean ± standard deviation ($\bar{x}\pm s$) and analyzed using the independent samples *t*-test; those not following a normal distribution were expressed as median and interquartile range [M (Q1, Q3)] and analyzed using the Mann–Whitney *U*-test, a non-parametric rank-sum test. A *P*-value < 0.05 was considered statistically significant.

### 2.8 Ethical considerations

This study was conducted in accordance with the Declaration of Helsinki and established ethical principles. All interview participants provided written informed consent prior to the interviews, and all Delphi expert panel members provided informed consent electronically before the initial questionnaire distribution. The research team has received training in data confidentiality, and all data will not be accessed by third parties. The study protocol was approved by the Ethics Committee of Yantai Affiliated Hospital of Shandong Medical And Pharmaceutical University (LW-20260507034).

## 3 Results

### 3.1 Literature search results

A total of 2,905 relevant records were identified through the database searches. After full-text screening, 16 articles (25–40) with strong relevance were included, comprising 9 quantitative studies, 5 qualitative studies, and 2 mixed-methods studies. The detailed screening process is illustrated in Figure 1. The basic characteristics of the included studies and the extracted content are summarized in Table 2.

**Figure 1:**
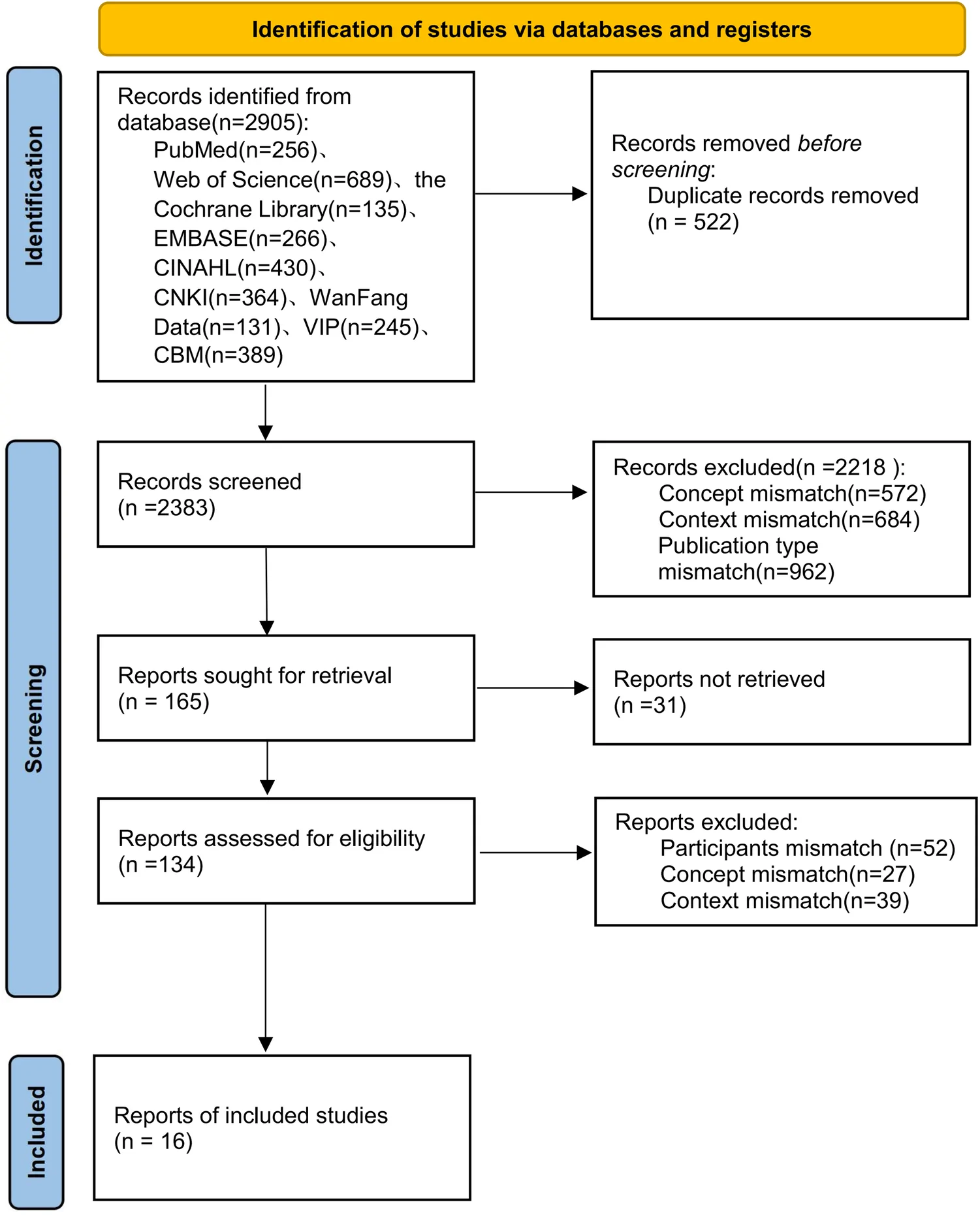
Flowchart of literature screening. CNKI, China National Knowledge Infrastructure; VIP, Weipu Data; CBM: the China Biology Medicine Database.

**Table 2:** Characteristics of the included articles (*n* = 16).

NO.	Author	Year	Country	Study Design	Theme	QQM Checklist
1	Liljeroos et al. (25)	2020	the Netherlands	Quantitative Study	Home-based social support services for patients with chronic heart failure should focus on psychological support and emotional monitoring to reduce the risk of hospital readmission.	High
2	Li et al. (26)	2025	China	Qualitative Study	Patients with chronic heart failure and their caregivers experience heavy burdens, negative emotions, and insufficient knowledge, necessitating multi-level social support ranging from physiological needs to self-actualization.	Moderate
3	Kamiya et al. (27)	2025	Japan	RCT	Home-based social support services for patients with chronic heart failure should leverage information and communication technology to share advance care plans, thereby addressing patients' needs for safety, respect, and belonging.	High
4	Olano-Lizarraga et al. (28)	2022	Spain	RCT	Home-based social support services for patients with chronic heart failure should emphasize joint participation by patients and their family members.	High
5	Kerwagen et al. (29)	2023	Germany	RCT	Remote management and mobile health technologies play a critical role in reducing mortality and hospital readmission rates in chronic heart failure.	High
6	Khajehpoor et al. (30)	2023	Iran	RCT	Home-based palliative care plays a pivotal role in improving the quality of life of patients with heart failure, with effects spanning psychological, familial, and socioeconomic dimensions.	High
7	Khera et al. (31)	2020	the United States	Quantitative Study	Insufficient medical insurance coverage and high out-of-pocket expenditures together constitute a financial burden for patients with cardiovascular diseases.	High
8	Camino Ortega et al. (32)	2023	Spain	Quantitative Study	Mobile health-based therapeutic education plays a key role in reducing readmission rates among patients with heart failure by enhancing patients' knowledge and self-management abilities.	High
9	Sun et al. (33)	2023	Japan	Quantitative Study	Home-care support clinics in Japan have played a critical role in reducing the risk of readmission among elderly patients with heart failure by providing 24-hour emergency visits and continuous medical care.	Moderate
10	Nordfonn et al. (34)	2020	Norway	Qualitative Study	Patients with heart failure develop self-management abilities amid treatment burdens through multi-dimensional support from healthcare providers, family members, and peers.	High
11	Siabani et al. (35)	2013	Australia	Qualitative Study	Social support services for patients with chronic heart failure need to comprehensively respond to their multi-level needs—from family belonging and safety assurance to cognitive empowerment.	High
12	Cheng et al. (36)	2024	China	Qualitative Study	Medication safety at home for patients with chronic heart failure is influenced by multiple factors, including knowledge, beliefs, self-regulation, and social facilitation.	High
13	Maddison et al. (37)	2025	Australia	Mixed-Methods Study	Smart home-based remote health monitoring, through multimodal data collection and personalized feedback, comprehensively addresses the social support needs of patients with chronic heart failure—from physiological surveillance to cognitive education.	High
14	Bartlett et al. (38)	2014	the United Kingdom	Mixed-Methods Study	Through personalized self-management systems that provide disease-related knowledge and behavioral support, patients with chronic heart failure can effectively enhance their cognitive level and promote changes in daily activity behaviors.	High
15	Durante et al. (39)	2023	Italy	Mixed-Methods Study	The multidimensional burden experienced by patients with heart failure suggests that social support services should systematically provide dyadic support—from patient-centered cognitive care to caregiver-focused psychological counseling.	High
16	Yang et al. (40)	2023	China	Mixed-Methods Study	Social support networks facilitate adherence to home-based cardiac rehabilitation exercise among patients with chronic heart failure by enhancing exercise self-efficacy and reducing fear of movement.	Moderate

### 3.2 Semi-Structured interview findings

#### 3.2.1 Baseline characteristics of interview participants

A total of 15 individuals participated in the interviews, comprising 8 patients with CHF and 7 caregivers of CHF patients. Detailed demographic and clinical characteristics of all participants are presented in Table 3.

**Table 3:** Demographic characteristics of the interview participants (*n* = 15).

Patients (*n* = 8)	Caregivers (*n* = 7)									
No.	Age (years)	Gender	NYHA Class	Admissions (n)	Disease Duration (years)	No.	Age (years)	Gender	Relationship to Patient	Caregiving Duration (years)
A1	52	Female	Ⅳ	3	4	B1	58	Male	Spouse	4
A2	78	Male	Ⅱ	2	5	B2	56	Female	Father–Daughter	5
A3	71	Male	Ⅳ	3	9	B3	46	Male	Father–Son	9
A4	70	Male	Ⅳ	2	8	B4	48	Male	Father–Son	6
A5	63	Female	Ⅳ	2	2	B5	38	Female	Mother–Daughter	2
A6	54	Female	Ⅲ	2	2	B6	63	Female	Spouse	9
A7	65	Male	Ⅳ	4	9	B7	55	Male	Spouse	2
A8	53	Male	Ⅲ	2	2					

#### 3.2.2 Thematic extraction from interview data

Content analysis was employed to analyze the interview data. The final themes extracted from the interviews are presented in Table 4. Based on the interview findings, combined with the results extracted from the literature, and following multiple rounds of discussion within the research group, a preliminary draft of the expert consultation framework for home-based social support service content for patients with chronic heart failure was constructed. This draft comprised 7 primary-level indicators, 17 secondary-level indicators, and 50 tertiary-level indicators, as detailed in Supplementary Table S3.

**Table 4:** Thematic framework of home-based social support needs in CHF patients.

Themes	Subthemes
A Physiological needs	A1 Housekeeping Services
	A2 Home-Based Professional Nursing Care
B Safety needs	B1 Home Physical Safety Support
	B2 Health Risk Monitoring Support Services
	B3 Medical Insurance and Reimbursement Support Services
	B4 Emergency Medical Support Services
C Love and belonging needs	C1 Core Caregiving and Time-Replacement Support Services
	C2 Psychological and Grief Counseling Support Services
	C3 Community and Peer Support Services
D Esteem needs	D1 Home-Based Palliative and Hospice Care Services
	D2 Advance Care Planning (ACP) Consultation Services
E Cognitive needs	E1 Disease-Related Health Education Support Services
	E2 Disease Self-Management Support Services
F Aesthetic needs	F1 Personal Image and Grooming Maintenance Support Services
	F2 Comfort Enhancement Support Services for the Healing Environment
G Self-actualization needs	G1 Social Role Reconstruction Support Services
	G2 Individualized Goal-Setting Support Services

### 3.3 Delphi expert consultation

#### 3.3.1 Baseline characteristics of experts

A total of 15 experts specializing in chronic heart failure and related fields were invited to participate, all of whom were based in China. The general characteristics of the experts are presented in Table 5.

**Table 5:** Demographic characteristics of the delphi expert panel (*n* = 15).

Category	Group	Number (n)	Percentage (%)
Gender	Male	5	33.3
	Female	10	66.7
Age	30–40	1	6.7
	41–50	11	73.3
	>50	3	20.0
Educational Level	Bachelor's	3	20.0
	Master's	5	33.3
	PhD	7	46.7
Professional Title	Intermediate	4	26.7
	Associate Senior	5	33.3
	Senior	6	40.0
Years of Working Experience	5–10	4	26.7
	11–20	8	53.3
	21–30	2	13.3
	>30	1	6.7
Research Area	Clinical Medicine	2	13.3
	Clinical Nursing	2	13.3
	Public Health	3	20.0
	Health Administration	2	13.3
	Chronic Disease Management	4	26.7
	Social Worker	2	13.3

#### 3.3.2 Expert positivity coefficient, authority coefficient, degree of consensus, and kendall's W

Fifteen questionnaires were distributed in each of the two consultation rounds, with a valid response rate of 100%, indicating a high level of expert engagement (Supplementary Table S4). The expert authority coefficients for the two rounds were 0.872 and 0.881, respectively, both exceeding 0.7 (Supplementary Table S5). Kendall's coefficients of concordance for the two rounds were 0.306 and 0.310 (*χ*^2^ = 335.588 and 356.368, respectively; both *P* < 0.001) (Table 6). The importance scores and coefficients of variation for indicators at all levels are detailed in Table 7.

**Table 6:** Kendall's W values for expert consensus across two delphi rounds.

Delphi Round	Indicator Level	Item Count	Kendall's W	*χ* ^2^	*P*
Round 1	First-Level Indicators	7	0.258	23.188	<0.001
	Second-Level Indicators	17	0.319	76.570	<0.001
	Third-Level Indicators	50	0.302	221.885	<0.001
	Total	74	0.306	335.588	<0.001
Round 2	First-Level Indicators	7	0.302	27.204	<0.001
	Second-Level Indicators	18	0.321	81.855	<0.001
	Third-Level Indicators	58	0.334	285.722	<0.001
	Total	83	0.310	356.368	<0.001

**Table 7:** Expert consensus on the home-based social support service content framework for CHF patients based on maslow's needs theory.

Indicator	Importance ($\bar{x}\pm s$)	Coefficient of Variation (CV)	Weight	Composite Weight	*λ*_max_	Consistency Ratio (CR)
A Physiological needs	5.000 ± 0.000	0.000	0.224		7.590	0.072
A1 Housekeeping Services	5.000 ± 0.000	0.000	0.500	0.112	2.000	0.000
A1.1 Home Environment Cleaning and Organizing Services	4.533 ± 0.640	0.141	0.116	0.013	5.157	0.035
A1.2 In-Home Bathing and Personal Hygiene Assistance Services	4.600 ± 0.507	0.110	0.179	0.020		
A1.3 Basic Meal Assistance Support	5.000 ± 0.000	0.000	0.374	0.042		
A1.4 Daily Errand and Life Affairs Assistance Services	4.467 ± 0.743	0.166	0.108	0.012		
A1.5 Basic Activities of Daily Living Services (including ambulation and positioning safety assistance, elimination care, and sleep care, etc.)	4.867 ± 0.352	0.116	0.223	0.025		
A2 Home-Based Professional Nursing Care	5.000 ± 0.000	0.000	0.500	0.112		
A2.1 Basic Nursing Care Support Services (e.g., temperature monitoring, skin care, oral care, oxygen therapy, and nebulization therapy, etc.)	4.800 ± 0.560	0.116	0.244	0.027	4.061	0.023
A2.2 Specialized Nursing Support Services (e.g., Internet-based nursing services for tube replacement, blood sampling and electrolyte monitoring, pressure ulcer risk assessment and management, and urinary catheterization, etc.)	4.860 ± 0.414	0.086	0.244	0.027		
A2.3 Heart Failure Symptom Management Support Services (e.g., refined management of dyspnea, activity–rest balance management, monitoring and dynamic assessment of body weight and edema signs, fluid intake–output management and precision nutritional support, and exercise rehabilitation and respiratory function training services, etc.)	5.000 ± 0.000	0.000	0.413	0.046		
A2.4 Specialized Device Management Support Services for Cardiac Function Support and Heart Failure Treatment (e.g., cardiac resynchronization therapy, left ventricular assist devices, and intra-aortic balloon pump counterpulsation, etc.)	4.533 ± 0.743	0.163	0.099	0.011		
B Safety needs	4.889 ± 0.323	0.066	0.089			
B1 Home Physical Safety Support	4.867 ± 0.352	0.072	0.217	0.019	4.060	0.023
B1.1 Home Functional Adaptability Modification Services	4.000 ± 0.756	0.188	0.089	0.002	4.061	0.023
B1.2 Safety Assistive Device Installation and Usage Guidance Services	4.533 ± 0.743	0.163	0.239	0.005		
B1.3 Home Safety Hazard Monitoring Services (e.g., formaldehyde detection, electrical appliance monitoring, and gas and oxygen leakage monitoring, etc.)	4.600 ± 0.632	0.137	0.433	0.008		
B1.4 Regular Environmental Disinfection and Infection Prevention and Control Guidance Services	4.533 ± 0.743	0.163	0.240	0.005		
B2 Health Risk Monitoring Support Services	4.933 ± 0.258	0.052	0.284	0.025		
B2.1 Intelligent Health Risk Early Warning Services (e.g., monitoring and early warning of health data through digital technologies such as wearable devices and mobile applications, etc.)	4.600 ± 0.632	0.137	0.667	0.017	2.000	0.000
B2.2 Internet-Plus Home Visit Services	4.533 ± 0.640	0.141	0.333	0.008		
B3 Medical Insurance and Reimbursement Support Services	4.600 ± 0.507	0.110	0.215	0.019		
B3.1 National Basic Medical Insurance Policy Consultation Services	4.733 ± 0.458	0.096	0.294	0.006	4.146	0.055
B3.2 Outpatient Chronic and Special Disease Qualification Determination and Reimbursement Process Support Services	4.467 ± 0.743	0.166	0.133	0.003		
B3.3 Critical Illness Insurance and Medical Assistance Comprehensive Consultation Services	4.400 ± 0.828	0.188	0.094	0.002		
B3.4 Commercial Supplementary Medical Insurance Consultation Support Services	5.000 ± 0.000	0.000	0.480	0.010		
B4 Emergency Medical Support Services	4.933 ± 0.258	0.052	0.284	0.025		
B4.1 Remote Online Consultation Services for Acute Heart Failure Symptoms	4.533 ± 0.834	0.183	0.312	0.008	3.054	0.052
B4.2 Emergency Coordination and Green Channel Services	4.467 ± 0.640	0.143	0.198	0.005		
B4.3 Multidisciplinary Heart Failure Team Cloud-Based Platform Support Services	4.600 ± 0.632	0.137	0.490	0.012		
C Love and belonging needs	4.944 ± 0.236	0.047	0.158			
C1 Core Caregiving and Time-Replacement Support Service	4.533 ± 0.640	0.141	0.141	0.022	3.054	0.052
C1.1 Daytime Care and Respite Support Services	4.200 ± 0.775	0.184	0.333	0.007	2.000	0.000
C1.2 Caregiver Respite Services	4.467 ± 0.834	0.186	0.667	0.015		
C2 Psychological and Grief Counseling Support Services	4.800 ± 0.414	0.086	0.334	0.053		
C2.1 Support Services for Building Harmonious Family Atmosphere and Relationships	4.733 ± 0.594	0.125	0.539	0.029	3.054	0.052
C2.2 Grief Counseling Support Services	4.667 ± 0.724	0.155	0.298	0.016		
C2.3 Anxiety/Depression Screening and Intervention Services	4.400 ± 0.828	0.188	0.164	0.009		
C3 Community and Peer Support Services	5.000 ± 0.000	0.000	0.525	0.083		
C3.1 Peer Support Services (e.g., community groups, patient support groups, etc.)	4.400 ± 0.737	0.167	0.143	0.012	3.000	0.000
C3.2 Community and Volunteer Organization Services	4.800 ± 0.775	0.161	0.429	0.036		
C3.3 Digital Platform-Based Social Interaction and Emotional Support Services	4.800 ± 0.775	0.161	0.429	0.036		
D Esteem needs	4.889 ± 0.323	0.066	0.109			
D1 Home-Based Palliative and Hospice Care Services	5.000 ± 0.000	0.000	0.486	0.053	3.018	0.018
D1.1 Independent Management of Daily Living Activities Services	4.333 ± 0.817	0.188	0.750	0.040	2.000	0.000
D1.2 Independent Social Participation Management Services	4.067 ± 0.799	0.196	0.250	0.013		
D2 Advance Care Planning (ACP) Consultation Services	4.333 ± 0.724	0.167	0.093	0.010		
D2.1 End-of-Life Symptom Control Services	4.933 ± 0.258	0.052	0.589	0.006	3.054	0.052
D2.2 Psychological and Spiritual Care Services	4.600 ± 0.632	0.137	0.159	0.002		
D2.3 Personalized Dignity Preservation and Culturally Tailored Care Services	4.667 ± 0.724	0.155	0.252	0.003		
D3 Advance Care Planning (ACP) Consultation Services	4.533 ± 0.743	0.163	0.421	0.046		
D3.1 Community-Based and Public Welfare ACP Consultation Support Services	4.400 ± 0.737	0.167	0.333	0.015	2.000	0.000
D3.2 ACP Documentation, Updating, and Archiving Services	4.533 ± 0.743	0.163	0.667	0.031		
E Cognitive needs	5.000 ± 0.000	0.000	0.224			
E1 Disease-Related Health Education Support Services	5.000 ± 0.000	0.000	0.500	0.112	2.000	0.000
E1.1 Heart Failure Specialist Outpatient Disease Awareness Intensive Education and Counseling Services	4.667 ± 0.617	0.132	0.159	0.018	3.054	0.052
E1.2 Community Health Service Center Chronic Disease Awareness Health Education Class Support Services	4.733 ± 0.594	0.125	0.252	0.028		
E1.3 Hospital–Community Collaborative Health Education Brochure and Video Push Services	5.000 ± 0.000	0.000	0.589	0.066		
E2 Disease Self-Management Support Services	5.000 ± 0.000	0.000	0.500	0.112		
E2.1 Standardized Heart Failure Medication Education Brochure and Video Push Services	4.467 ± 0.516	0.115	0.196	0.022	5.159	0.036
E2.2 Intelligent Medication Adherence Reminder Support Services	4.467 ± 0.640	0.143	0.196	0.022		
E2.3 Multi-Media Structured Education Support Services for Heart Failure Volume and Symptom Management	4.067 ± 0.704	0.173	0.072	0.008		
E2.4 Online Disease Management Programs for Heart Failure Patients and Their Family Caregivers	4.333 ± 0.817	0.188	0.133	0.015		
E2.5 Remote Health Monitoring Data Feedback and Guidance Services	4.800 ± 0.775	0.161	0.403	0.045		
F Aesthetic needs	4.444 ± 0.616	0.138	0.087			
F1 Personal Image and Grooming Maintenance Support Services	4.333 ± 0.617	0.142	0.320	0.028	2.000	0.000
F1.1 Beauty, Hairdressing, and Nail Care Support Services	4.133 ± 0.743	0.179	0.312	0.009	3.054	0.052
F1.2 Adaptive Dressing Guidance Services for Special Body Postures or Illness-Related Conditions	4.067 ± 0.704	0.173	0.198	0.006		
F1.3 Oral Hygiene and Breath Freshening Maintenance Support	4.267 ± 0.594	0.139	0.490	0.014		
F2 Comfort Enhancement Support Services for the Healing Environment	5.000 ± 0.000	0.000	0.680	0.059		
F2.1 Healing Environment Creation Services (e.g., lighting, color, and quietness, etc.)	4.467 ± 0.640	0.143	0.250	0.015	3.000	0.000
F2.2 Aesthetic Accessibility Home Environment Modification Services	4.467 ± 0.640	0.143	0.250	0.015		
F2.3 Home Environment Comfort Regulation Support Services	4.667 ± 0.617	0.132	0.500	0.030		
G Self-actualization needs	4.889 ± 0.323	0.066	0.109			
G1 Social Role Reconstruction Support Services	4.400 ± 0.828	0.188	0.200	0.022	2.000	0.000
G1.1 Family Role Restoration Support Services	4.067 ± 0.799	0.196	0.087	0.002	4.120	0.045
G1.2 Employment and Social Function Maintenance Support/Training Services	4.400 ± 0.828	0.188	0.166	0.004		
G1.3 Social Contribution Platform Development Services	4.467 ± 0.640	0.143	0.264	0.006		
G1.4 Personal Achievement and Life Review Services	4.600 ± 0.632	0.137	0.483	0.011		
G2 Individualized Goal-Setting Support Services	5.000 ± 0.000	0.000	0.800	0.088		
G2.1 Support Services for Rekindling and Re-exploring Hobbies and Interests	4.067 ± 0.799	0.196	0.106	0.010	3.054	0.052
G2.2 Support Services for Potential Discovery and Skill Enhancement	4.733 ± 0.458	0.096	0.544	0.048		
G2.3 Support Services for Creative Transformation of Interest-Based Achievements	4.600 ± 0.632	0.137	0.350	0.031		

#### 3.3.3 Revision and refinement of the indicator framework

Based on the indicator selection criteria and expert comments, revisions were made to the indicators following group discussions. In the first round of expert consultation, 3 tertiary-level indicators were deleted according to the selection criteria; based on expert feedback, an additional 2 tertiary-level indicators were removed, 11 tertiary-level and 2 secondary-level indicators were added, and 9 tertiary-level and 2 secondary-level indicators were modified. In the second round of expert consultation, 1 tertiary-level and 1 secondary-level indicator were modified, and 1 tertiary-level indicator was merged. The final indicator framework for home-based social support service content for patients with chronic heart failure comprised 7 primary-level, 18 secondary-level, and 58 tertiary-level indicators, as detailed in Table 7. Expert modification suggestions from the two Delphi rounds are presented in Supplementary Table S6.

## 4 Discussion

This study aimed to construct a content framework for home-based social support services for patients with chronic heart failure and represents an exploratory preliminary investigation in indicator system development. Its core output is an indicator set and weighting system derived from literature review, semi-structured interviews, and two rounds of Delphi expert consultation. It should be explicitly noted that this framework has not yet undergone field validation in real-world clinical settings. Its direct contribution lies in providing a theoretically driven needs indicator system with content validity evidence, rather than serving as a clinically validated intervention protocol or evaluation tool. The value of this study is positioned as furnishing a content foundation and theoretical framework for subsequent development of assessment instruments and intervention designs.

### 4.1 Scientific rigor and reliability of the content framework

Grounded in Maslow's hierarchy of needs, and informed by literature review, semi-structured interviews, and Delphi expert consultation, this study constructed a content framework for home-based social support services for patients with chronic heart failure, comprising 7 primary-level, 18 secondary-level, and 58 tertiary-level indicators. Maslow's hierarchy posits that individual needs follow a progressive sequence—that is, once basic physiological and safety needs are satisfied, individuals will further pursue higher-order needs such as love and belonging, esteem, and self-actualization (12). A total of 15 experts were enrolled in this study, covering multiple relevant fields including clinical medicine, clinical nursing, public health, health administration, chronic disease management, and social work. The experts had extensive work experience, with 11 (73.3%) having more than 10 years of practice; their educational backgrounds were also relatively advanced, with 12 (80%) holding a master's degree or above. The valid response rates for both rounds of expert consultation were 100%, and the expert authority coefficients were 0.872 and 0.881, respectively, both exceeding 0.7, indicating a high level of credibility in the consultation results. Kendall's W values for the two rounds were 0.306 and 0.310 (both *P* < 0.001), suggesting good consensus and convergence among expert opinions. Furthermore, the AHP was employed to determine the weight of indicators at each level, and the consistency ratios of all judgment matrices were below 0.10, indicating logical consistency and statistical acceptability of the weight assignments (41).

### 4.2 Analysis of the content framework based on Maslow's hierarchy of needs

#### 4.2.1 Support for physiological and safety needs as the foundational cornerstone of the framework

The results of this study show that the weight assigned to physiological needs support among the primary-level indicators was relatively high, suggesting that experts generally regard basic care and risk protection as the core components of home management for patients with chronic heart failure. This finding is closely related to the nature of chronic heart failure, as patients often experience dyspnea, fatigue, edema, decreased exercise tolerance, and recurrent acute exacerbations, resulting in sustained and frequent demands for daily living assistance, symptom monitoring, and professional nursing support throughout the disease trajectory (42). Older patients in particular often present with concurrent frailty, cognitive decline, and multimorbidity, necessitating external support to maintain basic functional capacity (43). Among the tertiary-level indicators, heart failure symptom management support services carried a relatively high composite weight, suggesting that dyspnea management, fluid volume monitoring, body weight observation, and guidance on balancing exercise and rest are critical components of home-based support. The 2024 Chinese Guidelines for the Diagnosis and Treatment of Heart Failure recommend that standardized symptom monitoring and volume management have been shown to reduce the risk of decompensation and decrease rehospitalization rates (44). The inclusion of relevant indicators in this framework aligns with such evidence-based recommendations.

Although the overall weight for safety needs support was relatively lower, certain subordinate indicators,such as health risk monitoring support services and emergency medical support services, carried relatively high composite weights, indicating that patients at home require not only daily safety protection but also medical accessibility and emergency care support. Patients with chronic heart failure experience marked fluctuations in their condition, and acute exacerbations can occur suddenly; if not recognized and addressed in a timely manner, they may rapidly progress to severe circulatory failure or even become life-threatening (45). Within the safety needs support dimension, indicators such as “intelligent health risk alert services,” “emergency medical support services,” and “multidisciplinary heart failure team cloud-platform support services” received high weights, suggesting that experts strongly endorse the importance of digital monitoring and multidisciplinary collaboration. Recent studies have shown that wearable devices, remote monitoring platforms, and multidisciplinary management models can significantly improve the efficiency of detecting heart failure deterioration, reduce unplanned hospitalizations, and improve patient outcomes (46, 47). Moreover, this study incorporated medical insurance reimbursement consultation, chronic/special disease qualification determination, and medical assistance into the safety needs domain, reflecting attention to the realities of China's healthcare environment. The financial burden imposed by long-term chronic disease treatment is a significant factor affecting patient adherence and healthcare utilization; economic support and policy accessibility are also essential components of home-based safety protection (11). Therefore, future development of home-based social support services for patients with chronic heart failure should expand from a purely medical care model to an integrated medical-care-policy tripartite support system.

#### 4.2.2 Multi-level needs support as an enrichment of the framework's connotation

The results of this study showed that, in addition to physiological and safety needs support, cognitive needs support and love-and-belonging needs support also carried relatively high weights among the primary-level indicators, suggesting that experts place increasing emphasis on multi-level needs. This finding indicates that the home-based support needs of patients with chronic heart failure are not limited to basic care, but also encompass multidimensional content such as disease-related knowledge, emotional support, and maintenance of social roles (48). From a theoretical perspective, Maslow's hierarchy emphasizes a progressive sequence from lower- to higher-order needs; however, in the context of long-term chronic disease management, patients often experience multiple needs simultaneously and interactively, rather than strictly emerging in a hierarchical order (49). The relatively high weights assigned to multi-level needs in this study suggest that, once basic care is largely met, patients' needs for disease knowledge, self-management, and emotional support gradually increase, aligning with the broader trend in chronic disease management from disease control toward comprehensive care (50).

At the specific dimensional level, the relatively high weight assigned to cognitive needs support suggests that patients have sustained and practical needs for disease-related knowledge acquisition and its application. Compared with Wang et al.'s (51) study on end-of-life home care needs among older adults with advanced chronic diseases, the present study revealed a higher weight for “cognitive needs,” which may be closely related to the nature of chronic heart failure. Patients with CHF are required to adhere to rigorous self-management over the long term, including fluid intake restriction, daily weight monitoring, low-sodium diet control, and early symptom recognition-behaviors that demand substantial disease-related knowledge reserves and cognitive executive capacity. Thus, the elevated weight of cognitive needs within the home-based support system for CHF reflects the objective shift in disease management from passive care toward active self-management. Previous studies have shown that health education is an important intervention for improving self-management capacity in CHF patients (52). However, in this study, indicators such as “remote health monitoring data feedback and guidance services” received relatively high weights, suggesting that patients are more concerned with dynamic feedback and individualized guidance than with unidirectional knowledge transmission. This finding indicates that current home-based health education models lack a continuous feedback mechanism, which may limit their effectiveness in promoting patients' self-management behaviors. Therefore, it is necessary to further integrate remote monitoring and feedback mechanisms on the basis of enhanced health education content, so as to improve patients' self-management capacity and long-term adherence.

Love and belonging needs support also occupied an important position in this study, with particularly high weight assigned to “community and peer support services,” suggesting that the model of emotional support is gradually extending from traditional intra-family provision toward a diversified social support network. Thapa et al. (53) reported that social support level is closely associated with psychological status and quality of life in patients with heart failure. Meanwhile, with shrinking family sizes and insufficient caregiving capacity, relying solely on family support often proves inadequate to meet patients' long-term care needs. Thus, building multi-level support networks through peer support, community resources, and digital platforms can help alleviate loneliness and enhance patients' capacity to cope with their condition. In addition, although the weight for self-actualization needs support was relatively lower, its connotation reflects a trend toward higher-level development in home-based care services. This study incorporated elements such as “life review” and “social role reconstruction,” extending the scope of chronic illness care. Previous studies have noted that patients with prolonged chronic conditions tend to experience diminished self-worth and weakened social roles (54). Theoretically, neglecting this dimension of needs may undermine patients' motivation and sustained engagement in disease management. Therefore, attending to patients' developmental needs is of great significance for promoting long-term self-management. Furthermore, this study included content related to aesthetic needs support, primarily grounded in patients' practical needs for living comfort, personal image maintenance, and home environmental experience in the context of long-term chronic illness. This reflects the extension of chronic care from disease management toward a focus on overall quality of life.

#### 4.2.3 Content validity and theoretical contributions of the framework

The content framework for home-based social support services for patients with chronic heart failure constructed in this study, guided by Maslow's hierarchy of needs, systematically integrates support contents corresponding to different levels of needs, demonstrating sound content validity in terms of theoretical completeness and coverage of need dimensions. The indicator contents of this framework were derived from dual sources of evidence, literature review and semi-structured interviews, and achieved consensus through two rounds of Delphi expert consultation, providing preliminary expert-level validation of its content validity. It should be emphasized that this study represents the construction and expert consensus phase of the content framework; all indicators require further feasibility and effectiveness validation in real-world clinical settings prior to application. Therefore, discussion of this framework at the current stage should focus on its content validity, that is, the extent to which the indicator contents systematically and comprehensively reflect the core need dimensions of home-based social support for CHF patients, and its face validity, namely the degree of expert consensus on the structural rationality and clinical acceptability of the framework. Whether this framework can reduce readmission rates, improve quality of life, enhance self-management adherence, or optimize self-care behaviors are all potential hypotheses based on theoretical logic, and await future longitudinal empirical studies for verification.

### 4.3 Adaptability of Maslow's hierarchy in the context of chronic illness: A hierarchical structure or a taxonomy of needs?

At the theoretical level, this study confronts an unavoidable conceptual tension: Maslow's hierarchy of needs is fundamentally predicated on a progressive sequence, positing that individuals pursue higher-order needs only after lower-order needs have been satisfied (12). However, in the context of long-term management of chronic heart failure, patients' physiological symptoms, psychological status, cognitive function, and social roles are often challenged simultaneously, with multiple need dimensions coexisting and interacting rather than emerging in a strict sequential order. This tension is also reflected in the findings of the present study-although physiological and safety needs ranked highest in weight, cognitive needs and love-and-belonging needs also carried relatively high weights, suggesting that the need structure of patients during the disease trajectory more closely resembles a multi-dimensional simultaneous pattern than a linear progressive one.

In response to this conceptual tension, the present study combined Maslow's hierarchy with the AHP, offering an operational pathway to address this issue. In a strictly hierarchical framework, lowe-order needs would inevitably take priority over higher-order needs in resource allocation; however, in the AHP weighting logic, “hierarchy” is operationalized as “quantified comparison of relative importance” rather than “temporal sequence of priority satisfaction.” In other words, indicators at all levels are compared in terms of their relative weights at the same time point, with experts determining the importance ranking of each dimension based on clinical experience and professional knowledge, rather than assuming that needs sequentially “emerge” or “disappear” over the disease course. This methodological choice allows the framework to retain the layered structure of Maslow's theory as an organizing dimension, while simultaneously enabling synchronized weighting and comprehensive evaluation of multiple coexisting needs in practice, thereby building a bridge between theoretical architecture and clinical reality.

Based on the above discussion, this study suggests that a more accurate characterization of this framework is as a chronic-disease-adapted transformation of Maslow's hierarchy, that is, a transition from a “strictly progressive hierarchical structure” to a “multi-dimensional need classification system based on importance ranking.” This transformation itself constitutes one of the theoretical contributions of this study, responding to prior critiques regarding the applicability of Maslow's theory in the chronic illness domain (49), and offering a referable modification pathway for future applications of this theory in chronic care.

## 5 Conclusion

This study, grounded in Maslow's hierarchy of needs and informed by literature review, semi-structured interviews, and two rounds of Delphi expert consultation, developed a content framework for home-based social support services for Chinese patients with chronic heart failure, comprising 7 primary-level, 18 secondary-level, and 58 tertiary-level indicators. Preliminary validation of the framework has been obtained in terms of content validity and face validity. Future research is needed to further validate the applicability and comprehensibility of the indicators among patient and caregiver populations, and to conduct feasibility pilots and longitudinal outcome evaluations so as to accumulate sufficient empirical evidence.

## 6 Limitations

This study has several limitations. First, the expert panel was predominantly recruited from China, which may limit the generalizability of the findings to other regions or healthcare systems. Second, the disciplinary composition of the expert panel did not include pharmacy, rehabilitation, or psychology, suggesting that the indicator system could be further supplemented by insights from these fields. Third, the operational feasibility and implementation effectiveness of the developed indicators require further validation in future studies.

## Data Availability

The original contributions presented in the study are included in the article/Supplementary Material, further inquiries can be directed to the corresponding author.
