# Supplementary material for: Construction of a home-based social support service content framework for Chinese patients with chronic heart failure based on Maslow's hierarchy of needs
Source: Front Med (Lausanne). 2026 Sep 21;13:1953127. doi: 10.3389/fmed.2026.1953127 (PMC13635349; doi:10.3389/fmed.2026.1953127)
Supplement: Supplementary file 1 [file Datasheet1.docx]

Supplementary Material

# **Supplementary Table 1 Search Strategies for English Databases**

| Databases | Search Strategies |
| --- | --- |
| PubMed | ("Heart Failure"[Mesh] OR "heart failure"[tiab] OR "cardiac failure"[tiab] OR "CHF"[tiab]) AND ("Social Support"[Mesh] OR "social support"[tiab] OR "emotional support"[tiab] OR "informational support"[tiab] OR "instrumental support"[tiab]) AND ("Home Care Services"[Mesh] OR "home care"[tiab] OR "home rehabilitation"[tiab] OR "home management"[tiab]) |
| Web of Science | TS=("heart failure" OR "cardiac failure" OR CHF) AND TS=("social support" OR "emotional support" OR "informational support" OR "instrumental support") AND TS=("home care" OR "home rehabilitation" OR "home management") |
| Cochrane | ("heart failure" OR "cardiac failure" OR CHF) AND ("social support" OR "emotional support" OR "informational support" OR "instrumental support") AND ("home care" OR "home rehabilitation" OR "home management") |
| EMBASE | ('heart failure'/exp OR 'heart failure':ti,ab OR 'cardiac failure':ti,ab OR chf:ti,ab) AND ('social support'/exp OR 'social support':ti,ab OR 'emotional support':ti,ab OR 'informational support':ti,ab OR 'instrumental support':ti,ab) AND ('home care'/exp OR 'home care':ti,ab OR 'home rehabilitation':ti,ab OR 'home management':ti,ab) |
| CINAHL | (MH "Heart Failure+" OR TI ("heart failure" OR "cardiac failure" OR CHF) OR AB ("heart failure" OR "cardiac failure" OR CHF)) AND (MH "Social Support+" OR TI ("social support" OR "emotional support" OR "informational support" OR "instrumental support") OR AB ("social support" OR "emotional support" OR "informational support" OR "instrumental support")) AND (MH "Home Care+" OR TI ("home care" OR "home rehabilitation" OR "home management") OR AB ("home care" OR "home rehabilitation" OR "home management")) |

# **Supplementary Table 2 Semi-Structured Interview Guide**

| Interviewees | Interview Content |
| --- | --- |
| **Patient** | ①In the context of home‑based rehabilitation, what are the primary difficulties you encounter in managing physiological symptoms—including dyspnea, edema, fatigue, dietary intake, and sleep? What specific types of support would you find most beneficial? |
|  | ②Regarding daily life at home, what are your primary concerns or perceived risks in safeguarding your health and personal safety? What guidance or assistance have you received to date? What additional support would you need to strengthen your sense of security and health protection in the home environment? |
|  | ③In your social interactions and relationships, what emotional challenges or interpersonal difficulties have you experienced? What forms of companionship, care, or assistance have you already received? What types of emotional support, peer companionship, or mutual aid services would you expect from family, community, or other organizations? |
|  | ④Throughout the care‑receiving process and daily living, have you encountered instances of being misunderstood, disrespected, or having your preferences overlooked? In what ways have family members or healthcare providers supported your autonomy and decision‑making? What further support would facilitate your ability to articulate your perspectives and actively engage in care‑related decisions? |
|  | ⑤With respect to home‑based daily life, what obstacles have you faced in acquiring disease‑specific knowledge, accessing health information, and learning self‑care strategies? What sources do you typically rely on for health information? What additional knowledge, skills, or informational resources would you like to obtain? |
|  | ⑥What are your expectations and needs regarding your living environment and personal presentation during home‑based daily life? What current limitations or sources of dissatisfaction exist? What further support would enhance your comfort, well‑being, and overall quality of life? |
|  | ⑦Since the onset of your illness, what transformations or challenges have you encountered in pursuing personal goals, fulfilling family roles, engaging in social activities, or sustaining a sense of self‑worth? What support have you already received in these domains? What additional assistance would you find valuable? |
| **Caregiver** | ①In the process of caring for the patient's daily life at home, what aspects of physical care do you find most challenging or stressful? What further support would you like to receive to better meet the care needs of both the patient and yourself? |
|  | ②During home-based care, what risks or hidden dangers do you perceive that may affect the health and safety of the patient or the family? What measures do you usually take to address these issues? What additional support or services do you think are needed to further enhance the safety and security of home-based care? |
|  | ③How has caregiving affected your relationships with the patient, family members, friends, or others? How do you usually cope with these impacts? What support or help would you like to receive from family, the community, healthcare institutions, or other social organizations to alleviate caregiving burden and gain more emotional support? |
|  | ④During the caregiving process, what difficulties have you encountered in expressing your opinions, needs, and thoughts? What support have you received so far? What help would you like to obtain to enhance your sense of being respected, recognized, and involved in relevant decision-making? |
|  | ⑤In the process of caring for the patient, what difficulties have you encountered in understanding disease-related knowledge, accessing health information, and learning caregiving skills? What guidance or help have you received? What additional knowledge, skills training, or informational support would you like to receive? |
|  | ⑥During the caregiving process, what needs or expectations do you have regarding the living environment, daily comfort, and your own quality of life? What further support would you like to receive to improve the comfort, pleasure, and overall quality of your home-based life? |
|  | ⑦Since taking on caregiving responsibilities, what changes or difficulties have you experienced in terms of family roles, work or study, social interactions, personal development, or realizing your self-worth? What further help would you like to receive to better balance caregiving responsibilities with personal development and achieve a sense of self-fulfillment? |
| Note: The order of the interview questions can be modified flexibly in response to the natural progression of the interview. | |

**Supplementary Table 3** **Preliminary Indicator Framework for Home-Based Social Support Services for Patients with Chronic Heart Failure (First Round Delphi Consultation)**

| First‑Level Indicators | Second‑Level Indicators | Third‑Level Indicators |
| --- | --- | --- |
| A Physiological needs | A1 Housekeeping Services | A1.1 Home Environment Cleaning and Organizing Services |
|  |  | A1.2 In‑Home Bathing and Personal Hygiene Assistance Services |
|  |  | A1.3 Basic Meal Assistance Support |
|  |  | A1.4 Daily Errand and Life Affairs Assistance Services |
|  | A2 Home‑Based Professional Nursing Care | A2.1 Basic Nursing Care Support Services (e.g., temperature monitoring, skin care, oral care, and nebulization therapy, etc.) |
|  |  | A2.2 Heart Failure Symptom Management Support Services (e.g., dyspnea, activity–rest balance, nutrition and diet, etc.) |
|  |  | A2.3 Specialized Device Management Support Services for Cardiac Function Support and Heart Failure Treatment (e.g., cardiac resynchronization therapy, left ventricular assist devices, and intra‑aortic balloon pump counterpulsation, etc.) |
|  |  | A2.4 Other Forms of Internet‑Plus Empowered Home‑Based Nursing Services |
|  |  | A2.5 Specialized Nursing Services (e.g., rehabilitation assistive device adaptation, tube replacement, and wound dressing change, etc.) |
| B Safety needs | B1 Home Physical Safety Support | B1.1 Comprehensive Age‑Friendly Home Modification Services |
|  |  | B1.2 Safety Assistive Device Installation and Usage Guidance Services |
|  |  | B1.3 Home Safety Hazard Monitoring Services (e.g., formaldehyde detection, electrical appliance monitoring, and gas leakage monitoring, etc.) |
|  | B2 Health Risk Monitoring Support Services | B2.1 Wearable Health Monitoring Device Usage Support Services |
|  |  | B2.2 Mobile Health Application Usage Services |
|  |  | B2.3 Internet‑Plus Home Visit Services |
|  | B3 Medical Insurance and Reimbursement Support Services | B3.1 National Basic Medical Insurance Policy Consultation Services |
|  |  | B3.2 Outpatient Chronic and Special Disease Qualification Determination and Reimbursement Process Support Services |
|  |  | B3.3 Critical Illness Insurance Reimbursement Policy Consultation Support Services |
|  |  | B3.4 Commercial Supplementary Medical Insurance Consultation Support Services |
|  | B4 Emergency Medical Support Services | B4.1 Remote Online Consultation Services for Acute Heart Failure Symptoms |
|  |  | B4.2 Green Channel Services for Acute Heart Failure Exacerbation |
|  |  | B4.3 Multidisciplinary Heart Failure Team Cloud‑Based Platform Support Services |
| C Love and belonging needs | C1 Core Caregiving and Time‑Replacement Support Service | C1.1 Daytime Care and Respite Support Services |
|  |  | C1.2 Caregiver Respite Services |
|  | C2 Psychological and Grief Counseling Support Services | C2.1 Support Services for Building Harmonious Family Atmosphere and Relationships |
|  |  | C2.2 Grief Counseling Support Services |
|  | C3 Community and Peer Support Services | C3.1 Peer Support Services (e.g., community groups, patient support groups, etc.) |
|  |  | C3.2 Community and Volunteer Organization Services |
|  |  | C3.3 Digital Platform‑Based Social Interaction and Emotional Support Services |
| D Esteem needs | D1 Home‑Based Palliative and Hospice Care Services | D1.1 End‑of‑Life Symptom Control Services |
|  |  | D1.2 Psychological and Spiritual Care Services |
|  |  | D1.3 End‑of‑Life Preparation and Hospice Care Services |
|  | D2Advance Care Planning (ACP) Consultation Services | D2.1 Community‑Based and Public Welfare ACP Consultation Support Services |
|  |  | D2.2 ACP Documentation, Updating, and Archiving Services |
| E Cognitive needs | E1 Disease‑Related Health Education Support Services | E1.1 Heart Failure Specialist Outpatient Disease Awareness Intensive Education and Counseling Services |
|  |  | E1.2 Community Health Service Center Chronic Disease Awareness Health Education Class Support Services |
|  |  | E1.3 Hospital–Community Collaborative Health Education Brochure and Video Push Services |
|  | E2 Disease Self‑Management Support Services | E2.1 Standardized Heart Failure Medication Education Brochure and Video Push Services |
|  |  | E2.2 Intelligent Medication Adherence Reminder Support Services |
|  |  | E2.3 Structured Learning Module Support Services for Heart Failure Volume/Symptom Management via App |
|  |  | E2.4 Structured Educational Materials Support Services for Self‑Monitoring Skills  E2.5 Online Disease Management Programs for Heart Failure Patients and Their Family Caregivers |
| F Aesthetic needs | F1 Personal Image and Grooming Maintenance Support Services | F1.1 Beauty, Hairdressing, and Nail Care Support Services |
|  |  | F1.2 Adaptive Dressing Guidance Services for Special Body Postures or Illness‑Related Conditions |
|  | F2 Comfort Enhancement Support Services for the Healing Environment | F2.1 Healing Environment Creation Services (e.g., lighting, color, and quietness, etc.) |
|  |  | F2.2 Aesthetic Accessibility Home Environment Modification Services |
| G Self-actualization needs | G1 Social Role Reconstruction Support Services | G1.1 Family Role Restoration Support Services |
|  |  | G1.2 Employment and Social Function Maintenance Support/Training Services |
|  | G2 Individualized Goal‑Setting Support Services | G2.1 SMART‑Based Individualized Rehabilitation Goal‑Setting Services |
|  |  | G2.2 Individualized Goal Implementation Progress Follow‑Up Services |

**Supplementary Table 4 Questionnaire Recovery Status Across Two Delphi Rounds**

| Round | Distributed (n) | Valid Returns (n) | Effective Response Rate (%) | Experts with Suggestions (n) | Expert Suggestion Rate (%) |
| --- | --- | --- | --- | --- | --- |
| 1 | 15 | 15 | 100% | 8 | 53.4% |
| 2 | 18 | 18 | 100% | 3 | 20.0% |

**Supplementary Table 5 Authority Coefficients (Cr) of Delphi Experts Across Two Consultation Rounds**

| Round | Expert No. | Basis for Judgment | Familiarity with the Topic | Expert Authority Coefficient (Cr) |
| --- | --- | --- | --- | --- |
| 1 | 1 | 1.0 | 0.8 | 0.9 |
|  | 2 | 0.9 | 0.8 | 0.9 |
|  | 3 | 0.9 | 0.9 | 0.9 |
|  | 4 | 0.9 | 0.8 | 0.9 |
|  | 5 | 1.0 | 1.0 | 1.0 |
|  | 6 | 1.0 | 0.8 | 0.9 |
|  | 7 | 0.8 | 1.0 | 0.9 |
|  | 8 | 0.8 | 0.8 | 0.8 |
|  | 9 | 0.8 | 0.8 | 0.8 |
|  | 10 | 1.0 | 0.8 | 0.9 |
|  | 11 | 0.9 | 0.8 | 0.9 |
|  | 12 | 0.9 | 0.8 | 0.9 |
|  | 13 | 0.8 | 0.8 | 0.8 |
|  | 14 | 0.9 | 0.8 | 0.9 |
|  | 15 | 0.9 | 0.8 | 0.9 |
| 2 | 1 | 1.0 | 0.8 | 0.9 |
|  | 2 | 1.0 | 0.8 | 0.9 |
|  | 3 | 0.8 | 1.0 | 0.9 |
|  | 4 | 0.9 | 0.8 | 0.9 |
|  | 5 | 1.0 | 1.0 | 1.0 |
|  | 6 | 1.0 | 0.8 | 0.9 |
|  | 7 | 0.8 | 1.0 | 0.9 |
|  | 8 | 0.8 | 0.8 | 0.8 |
|  | 9 | 0.8 | 0.8 | 0.8 |
|  | 10 | 1.0 | 0.8 | 0.9 |
|  | 11 | 0.9 | 0.8 | 0.9 |
|  | 12 | 0.9 | 0.8 | 0.9 |
|  | 13 | 0.8 | 0.8 | 0.8 |
|  | 14 | 0.9 | 0.8 | 0.9 |
|  | 15 | 0.8 | 1.0 | 0.9 |

**Supplementary Table 6	Delphi Expert Consultation Comment**

| Round | Comments | |
| --- | --- | --- |
| 1 | Deletion of 5 second-level indicators | “Structured Educational Materials Support Services for Self‑Monitoring Skills” |
|  |  | “SMART‑Based Individualized Rehabilitation Goal‑Setting Services” |
|  |  | “Individualized Goal Implementation Progress Follow‑Up Services” |
|  |  | “Dignity Communication and Psychological Support Training Services” |
|  |  | “Dignity Maintenance Training Services in Daily Care” |
|  | Addition of 2 second‑level indicators | “Home‑Based Independent Decision‑Making Support Services” |
|  |  | “Hobbies, Interests, and Potential Development Support” |
|  | Addition of 13 third‑level indicators | “Basic Activities of Daily Living Services (including ambulation and positioning safety assistance, elimination care, and sleep care, etc.)” |
|  |  | “Intelligent Health Risk Early Warning Services” |
|  |  | “Anxiety/Depression Screening and Intervention Services” |
|  |  | “Independent Management of Daily Living Activities Services” |
|  |  | “Independent Social Participation Management Services” |
|  |  | “Remote Health Monitoring Data Feedback and Guidance Services” |
|  |  | “Oral Hygiene and Breath Freshening Maintenance Support” |
|  |  | “Social Contribution Platform Development Services” |
|  |  | “Personal Achievement and Life Review Services” |
|  |  | “Support Services for Rekindling and Re‑exploring Hobbies and Interests” |
|  |  | “Support Services for Potential Discovery and Skill Enhancement” |
|  |  | “Support Services for Creative Transformation of Interest‑Based Achievements” |
|  |  | “Regular Environmental Disinfection and Infection Prevention and Control Guidance Services” |
|  | Merger of 1 third‑level indicator | “Structured Learning Module Support Services for Heart Failure Volume/Symptom Management via App” and “Structured Educational Materials Support Services for Self‑Monitoring Skills” were merged into: → “Multi‑Media Structured Education Support Services for Heart Failure Volume and Symptom Management” |
|  | Modification of 2 second‑level indicators | “Psychological and Grief Counseling Support Services” → modified to “Comprehensive Life‑Course Psychological Support Services” |
|  |  | “Social Role Reconstruction Support Services” → modified to “Sense of Value and Social Role Reconstruction Support Services” |
|  | Modification of 7 third‑level indicators | “Basic Nursing Care Support Services (e.g., temperature monitoring, skin care, oral care, and nebulization therapy, etc.)” → modified to “Basic Nursing Care Support Services (e.g., temperature monitoring, skin care, oral care, oxygen therapy, and nebulization therapy, etc.)” |
|  |  | “Heart Failure Symptom Management Support Services (e.g., dyspnea, activity–rest balance, nutrition and diet, etc.)” → modified to “Specialized Nursing Support Services (e.g., Internet‑based nursing services for tube replacement, blood sampling and electrolyte monitoring, pressure ulcer risk assessment and management, and urinary catheterization, etc.)” |
|  |  | “Specialized Device Management Support Services for Cardiac Function Support and Heart Failure Treatment (e.g., cardiac resynchronization therapy, left ventricular assist devices, and intra‑aortic balloon pump counterpulsation, etc.)” → modified to “Heart Failure Symptom Management Support Services (e.g., refined management of dyspnea, activity–rest balance management, monitoring and dynamic assessment of body weight and edema signs, fluid intake–output management and precision nutritional support, and exercise rehabilitation and respiratory function training services, etc.)” |
|  |  | “Comprehensive Age‑Friendly Home Modification Services” → modified to “Home Functional Adaptability Modification Services” |
|  |  | “Critical Illness Insurance Reimbursement Policy Consultation Support Services” → modified to “Critical Illness Insurance and Medical Assistance Comprehensive Consultation Services” |
|  |  | “Green Channel Services for Acute Heart Failure Exacerbation” → modified to “Emergency Coordination and Green Channel Services” |
|  |  | “End‑of‑Life Preparation and Hospice Care Services” → modified to “Personalized Dignity Preservation and Culturally Tailored Care Services” |
| 2 | Merger of 1 third‑level indicator | “Wearable Health Monitoring Device Usage Support Services” and “Mobile Health Application Usage Services” were merged into: → “Intelligent Health Risk Early Warning Services (e.g., monitoring and early warning of health data through digital technologies such as wearable devices and mobile applications, etc.)” |
|  | Modification of 1 second‑level indicator | “Core Caregiving and Time‑Replacement Support Services” → modified to “Compassionate Care and Companion Support Services” |
|  | Modification of 1 third‑level indicator | “Psychological and Spiritual Care Services” → modified to “Psychological and Spiritual Care Services” |
